# Vessel size as a marker of survival in estrogen receptor positive breast cancer

**DOI:** 10.1007/s10549-023-06974-4

**Published:** 2023-05-24

**Authors:** Vladan Milosevic, Reidunn J. Edelmann, Ingeborg Winge, Carina Strell, Artur Mezheyeuski, Gøril Knutsvik, Cecilie Askeland, Elisabeth Wik, Lars A. Akslen, Arne Östman

**Affiliations:** 1 Centre for Cancer Biomarkers CCBIO, Department of Clinical Medicine, University of Bergen, Bergen, Norway; 2 Department of Pathology, Haukeland University Hospital, Bergen, Norway; 3 Department of Immunology, Genetics and Pathology, Uppsala University, Uppsala, Sweden; 4 Department of Oncology and Pathology, Karolinska Institutet, Solna, Sweden

**Keywords:** Angiogenesis, Alpha-SMA, CD34

## Abstract

**Purpose:**

Angiogenesis is crucial for tumor growth and is one of the hallmarks of cancer. In this study, we analyzed microvessel density, vessel median size, and perivascular a-SMA expression as prognostic biomarkers in breast cancer.

**Methods:**

Dual IHC staining was performed where alpha-SMA antibodies were used together with antibodies against the endothelial cell marker CD34. Digital images of stainings were analyzed to extract quantitative data on vessel density, vessel size, and perivascular alpha-SMA status.

**Results:**

The analyses in the discovery cohort (*n* = 108) revealed a statistically significant relationship between large vessel size and shorter disease-specific survival (*p* = 0.007, log-rank test; *p* = 0.01, HR 3.1; 95% CI 1.3–7.4, Cox-regression analyses). Subset analyses indicated that the survival association of vessel size was strengthened in ER + breast cancer. To consolidate these findings, additional analyses were performed on a validation cohort (*n* = 267) where an association between large vessel size and reduced survival was also detected in ER + breast cancer (*p* = 0.016, log-rank test; *p* = 0.02; HR 2.3, 95% CI 1.1–4.7, Cox-regression analyses).

**Conclusion:**

Alpha-SMA/CD34 dual-IHC staining revealed breast cancer heterogeneity regarding vessel size, vessel density, and perivascular a-SMA status. Large vessel size was linked to shorter survival in ER + breast cancer.

**Supplementary Information:**

The online version contains supplementary material available at 10.1007/s10549-023-06974-4.

## Introduction

Angiogenesis is crucial for tumor growth when the tumor size is exceeding 1–2 mm^3^, and it is one of the hallmarks of cancer [1–4]. Upon neovascularization, tumor growth speeds up and becomes exponential [4, 5]. Newly grown vessels provide the necessary nutrients and oxygen to support the increased tumor growth, but they are usually immature and both morphologically and physiologically aberrant.

Tumor vessels are exerting several pathological features such as the formation of irregular vascular networks, loss of proper architecture, increased permeability, and low pericyte coverage [6–10]. The aberrant vessel structure is associated with dysfunctional blood flow, perpetuates extravasation of cancer cells, facilitates metastatic processes, and thus, contributes to tumor progression and aggressiveness [11].

Quantitative evaluation of tumor neo-angiogenesis has been proposed as a valid method to assess the disease prognosis [12]. In the past few decades, several features and measurements of tumor angiogenesis have been proposed as potential prognostic tissue-based markers [12, 13]. Microvessel density (MVD) and improved measurements that better reflect ongoing angiogenesis such as proliferating MVD (pMVD) together with Vascular Proliferation Index (VPI), have been suggested as estimates of angiogenesis and showed validity as prognostic biomarkers [14–20]. A series of studies have also explored the prognostic significance of endothelial markers associated with specific vessel phenotypes [21–24].

Pericytes represent a heterogeneous population of cells that take part in building the mural layer of small vessels [25]. Being embedded in the basal membrane and also in close contact with endothelial cells, they take part in paracrine communication with endothelial cells and play an important role in vessel maturation, endothelial cell survival, vessel wall stabilization, and blood flow normalization [26–29]. Normally, in healthy tissues, pericytes express markers such as neural\glial antigen 2 (NG2), desmin, alpha-smooth muscle actin (a-SMA), and PDGFRb [reviewed in 25] [30]. Various prognosis associations have been detected based on perivascular marker status and have been associated with prognosis in studies done on, e.g., renal carcinoma, oral squamous cell carcinoma, NSCLC, endometrial cancer, and colorectal cancer [15, 31–35]. In a phase II study done on breast cancer patients in order to investigate potential mechanisms of bevacizumab neoadjuvant therapy benefits in certain subsets of breast cancer, a-SMA coverage was used as an indicator of vascular maturity and vascular normalization after bevacizumab treatment and was considered an indicator of favorable therapy response in patients [36].

In this study, we evaluate the prognostic value of vessel median size, perivascular a-SMA expression and baseline MVD in breast cancer. Vessel median size was included as a marker since this feature remains not commonly analyzed and could act as a proxy for vessel maturation. By digital reading of vascular markers in a population-representative cohort and validation in an independent series, we assessed how these vascular markers perform as independent prognosticators in breast cancer.

## Materials and methods

### Patients and tissue material

This study included cancer tissue from 439 breast cancer patients (108 cases as whole section slides, and 331 cases as TMA slides) (Supplementary Table S1). The material was obtained from women diagnosed with breast cancer in the period between 1996 and 2003, as part of the population-based Norwegian Breast Cancer Screening Program. These patients received treatment according to standard national protocols for that time at Haukeland University Hospital. Clinicopathologic data were available at the institution from the clinical records and histopathology reports in addition to microscopic re-examination. The follow-up information was collected from the Norwegian Cause of Death Registry. The last date of follow-up was June 30, 2017 and is considered complete and accurate. Collected outcome data consisted of survival status, survival time, and cause of death. The clinicopathologic data included age at diagnosis, the largest tumor diameter, histologic grade, molecular subtype, lymph node status, and immunohistochemical markers: hormonal receptors and HER2. Patient records and personal information were anonymized prior to analysis. A more detailed description of this cohort and clinical characteristics is available in the study by Knutsvik et al. [37]. Written informed consent has been obtained from all of the patients. The study was approved by the Western Regional Committee for Medical and Health Research Ethics, REC West (REK 2014/1984). All studies were performed in accordance with guidelines and regulations by the University of Bergen and REK, and in accordance with the Declaration of Helsinki Principles.

Obtained tissue samples were fixed using 4% buffered formaldehyde before further processing and embedding in the paraffin blocks. From the collected blocks, 5 μm sections were made using the same microtome and by the same operator and mounted on the poly-lysine-coated glass slides. Slides were kept at + 4 °C until antibody staining.

For the staining performed on the whole section slides, the hematoxylin–eosin-stained breast cancer tissues were first examined by an experienced pathologist, and representative tissue blocks from each case, containing both peripheral and central parts of the tumor, and with the most cellular and high-grade areas, were selected for staining. Selected paraffin blocks were also used in the preparation of tissue microarrays (TMA). TMAs were made with the help of hematoxylin–eosin-stained slides of corresponding paraffin blocks in order to select areas of high tumor purity and to include the tumor periphery. Cores were made in triplicate from each of the blocks, by punching cores of 1 mm in diameter and mounting them into the recipient paraffin block using a semi-automated precision instrument (Minicore 3, Tissue Arrayer, Alphelys, France).

### Immunohistochemistry staining

Immunohistochemistry was performed on 5-μm-thick breast cancer tissue sections made from formalin-fixed and paraffin-embedded archival tumor tissue, prepared in a form of whole section slides and TMA slides. Prior to IHC staining, slides were baked in the oven at 60 °C for 48 h. Following the baking step, slides were pretreated on the Ventana Discovery Ultra platform (Ventana Medical Systems Inc. Tucson, Arizona, USA; Roche diagnostics GmbH, Manheim, Germany). During the Ventana protocol, anti a-SMA antibody (M0851, Dako, clone 1A4, 1:2000) and secondary, AP multimer anti-mouse antibody (UltraMap—anti-Ms AP, 760-4312, Ventana Medical Systems Inc. Tucson, Arizona, USA; Roche diagnostics GmbH, Manheim, Germany, provided prediluted) were automatically applied by Ventana and color was developed using ChromoMap Blue chromogen kit (760-161, Ventana Medical Systems Inc. Tucson, Arizona, USA; Roche diagnostics GmbH, Manheim, Germany). After processing the slides in Ventana, slides were collected and cleaned with the detergent in order to remove the remaining liquid coverslip (LCS, 650-010, Ventana Medical Systems Inc. Tucson, Arizona, USA; Roche diagnostics GmbH, Manheim, Germany). Following this, slides were blocked for 10 min at RT in a humidity chamber using DAKO protein block (Protein Block Serum-Free Ready-To-Use Dako). Slides were then incubated with anti-CD34 antibody (M7165, Dako, clone QBend10, 1:80) overnight at 4 °C in a humidity chamber. The slides were washed three times for 5 min, using Dako washing buffer (S3006, Dako-Agilent, Copenhagen, Denmark), and incubated with secondary AP polymer-conjugated anti-mouse antibody (MP-5402, polyclonal, Vector Laboratories, Burlingame, USA, provided prediluted) for 1 h at RT in a humidity chamber. After washing (3 × 5 min) using Dako washing buffer (S3006, Dako-Agilent, Copenhagen, Denmark), color was developed using liquid permanent red (LPR) as chromogen (K0640, Dako-Agilent, Copenhagen, Denmark) for 5 min at RT. Thereafter, slides were rinsed with distilled water and then dehydrated using the increasing concentration of ethanol and then baked in the oven at 60 °C for 1 h to stabilize the LPR staining. After the baking step, slides were incubated in xylene for 5 min, and then the cover glass was mounted using an automated mounting machine (CoverSliper CR100, Dako-Agilent, Copenhagen, Denmark). An example of positive and negative control staining is shown in Supplementary Fig. S1.

### Digital image analysis

The double-stained slides were scanned using NanoZoomer-XR (Hamamatsu Photonics K.K., Shizuoka, Japan) with × 40 objective. To visualize the scanned files, Aperio ImageScope 12.4.3.5008 software was used. Individual images corresponding to each TMA core were extracted using QuPath software and saved in.tiff format. Before the image analysis was performed, every image was inspected by an experienced pathologist in order to assess the overall staining quality, tissue, and scanning quality. All the viable tumor tissue was used in the study. Some of the images needed to undergo manual curation in order to exclude artifacts, large areas of fat tissue, fibrosis, necrotic areas, and areas with benign tissue. Each case was represented by three cores from the tumor. If the TMA core failed the quality control, the sample was excluded from further analysis. The cores of the same origin were treated for image analysis as one entire tissue sample. Images that fulfilled the quality criteria were analyzed using Image J software. Regions of interest (ROI) on the whole section slides were manually annotated by a pathologist using QuPath software and processed using Image J software.

CD34 staining was used alongside an in-house developed image analysis algorithm, including size filtering and not counting single CD34 + cells, to identify vessels, as previously described [38, 39]. Specifically, the RGB image was subjected to a color deconvolution algorithm, and 256-grade layer was generated representing specific CD34 + staining. The intensity threshold was selected based on visual evaluation and applied to binarize the image. Next, the median filter with effective radius = 2 pixels was applied to remove small objects which are likely to be noise or nonspecific staining. Several approaches were used to address the potential situations with incomplete vessel staining, staining with brakes, and stained objects with holes, i.e., object enlargement, followed by hole filling command, application of mean and median filters (effective radius = 2 pixels), and object shrinking to original size. These image-processing actions resulted in the removal of non-vessel staining (weak background staining, unspecific chromophore precipitation, potential non-vessel CD34 + staining). Vessel size was determined as minimal Feret diameter (minimal distance between two parallel tangents of the analyzed object). The decision to use minimal Feret diameter was made to avoid potential alterations of visible vessel size due to non-tangential cut, when performing sample sectioning. The minimal Feret diameter of the smallest identified vessels was 4 um, which is well-corresponding to the characteristic capillary size of 5–8 um in vivo and considering its deformation during sample handling and fixation.

Vessel identification was used to determine metrics such as vessel density (calculated as a number of detected vessels per total sample area normalized to the area of 1 mm^2^), to assess the vessel diameter (measured as a minimal Feret diameter of each vessel and summarized as a median value across all vessels of each case) and to identify perivascular spaces. Perivascular space was defined as the area surrounding each CD34 positive structure (vessel) at a 10-pixel distance. After determining the perivascular region, the a-SMA staining intensity levels were assessed using an in-house developed image analysis algorithm (Image J software), as described previously in detail [38]. In short, a-SMA expression was evaluated after color deconvolution by transforming the pixel intensity of the staining into optical density (OD) using the equation *OD* = Log10(I_0_/I), where I represents the pixel intensity and I_0_ is the maximal possible pixel intensity. As in the digital images pixel values are integers ranging from 0 (complete black) to 255 (complete white), 255 represents the complete absence of staining and 0 represents the complete absence of transmitted light or the maximal staining intensity (*I*_max_ = I_0_). In this way, calculated OD value is linearly associated with the staining intensity. The output data are generated as a median value for each individual core and for each case. In the case of whole section slides, the individual sample images were representing one tissue piece and were treated as a case. a-SMA staining outside perivascular regions, possibly derived from cancer-associated fibroblasts, was not included in digital scoring.

Analyses generated the following tissue metrics: stromal area per mm^2^ (the area of marker defined stroma), stromal a-SMA intensity (the median value of the stromal a-SMA optical density across all images representing each case), perivascular a-SMA intensity (PVI—the median value of the a-SMA optical density measured in perivascular space across all images representing one case) and a fraction of a-SMA covered vessels (FCV—the median value of a-SMA covered vessels fraction calculated per case by classifying vessels as “covered” or “uncovered” based on the median perivascular intensity of marker expression as a cut-off) and aforementioned vessel density and vessel median diameter. The computation of tissue metrics was performed assuming the image resolution is 4 pix/um. All measurements were performed in the intra-tumor regions.

### Statistical analysis and REMARK criteria

The Spearman two-tailed test was used to determine the correlation between continuous variables. The Spearman correlation coefficient higher/lower than ± 0.5 and a *p* value < 0.01 were considered as threshold reference values. For determining associations between different clinicopathologic subsets and tissue metrics presented as continuous data, Mann–Whitney and Kruskal–Wallis tests were used. For survival analysis, continuous variables were first dichotomized based on their median values, and Kaplan–Meier curves with the log-rank test were then made, with the p value cutoff for statistical significance set at 0.05. In order to calculate hazard ratios of the clinicopathologic factors and tissue metrics for patient survival, Cox-regression analysis was performed on dichotomized values with both univariable and multivariable settings, with the *p* value cutoff for statistical significance set at 0.05. Dichotomization on “low (0–50%)” group and “high (> 50%)” group was made at the median.

All tests were performed using SPSS version 26 (SPSS Inc., Chicago, IL). Forest Plot was designed in Microsoft Office Excel 365 (Microsoft Inc., Redmond, WA, USA).

As indicated in the Supplementary Table S13, study was performed according to the REMARK criteria.

## Results

### a-SMA and CD34 double staining reveal breast cancer heterogeneity regarding vessel size, perivascular a-SMA status, and vessel density

Dual IHC stainings were performed where a-SMA antibodies were combined with antibodies detecting the endothelial cell marker CD34. Analyses were performed on whole section slides from 108 cases of a population-based cohort of mammography-detected breast cancers. Case characteristics are summarized in Supplementary Table S1. Digital images of stainings were analyzed to extract quantitative data on vessel density, vessel size, and perivascular a-SMA status (see Material and Methods for details).

**Fig. 1:**
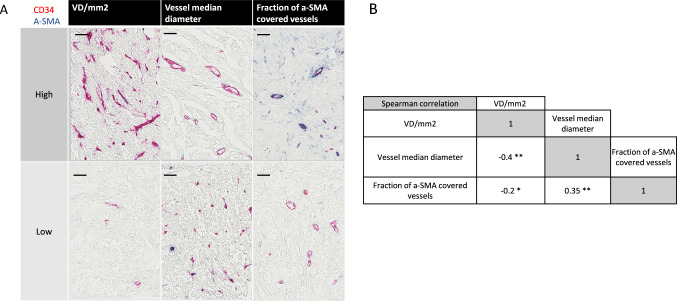
**A** Intertumoral heterogeneity in vessel median size, vessel density, and fraction of a-SMA covered vessels in the “whole section” cohort. Double staining with CD34 and a-SMA showing examples of high and low cases regarding vessel size, vessel density, and fraction of a-SMA covered vessels between different cases in the “whole section” cohort. Variables were dichotomized as “low (0–50%)” group and “high (> 50%)” group, based on the median value. Size bars correspond to 100 μm. **B** Intertissue metric correlations. Spearman correlation coefficients representing associations between vessel median size, vessel density, and fraction of a-SMA covered vessels in the “whole section” cohort. *p < 0.05, **p < 0.001

The initial analysis demonstrated large inter-case heterogeneity between the tumors regarding all three metrics. (Fig. 1A). Correlation analyses of the three metrics revealed positive correlations between vessel size and the fraction a-SMA-covered vessels. In contrast, negative correlations were detected between vessel density and both vessel size and fraction a-SMA-covered vessels (Fig. 1B).

### Vessel features are not associated with clinicopathologic characteristics, but large vessel size is linked to shorter survival

The quantitative data from the vascular profiling were used to investigate potential associations between these features and standard clinicopathologic characteristics.

As shown in Supplementary Tables S2–S4, none of the three vascular metrics displayed any associations with age, tumor size, histological grade, lymph node status, ER/PR/HER2 status, or molecular subtype.

**Fig. 2:**
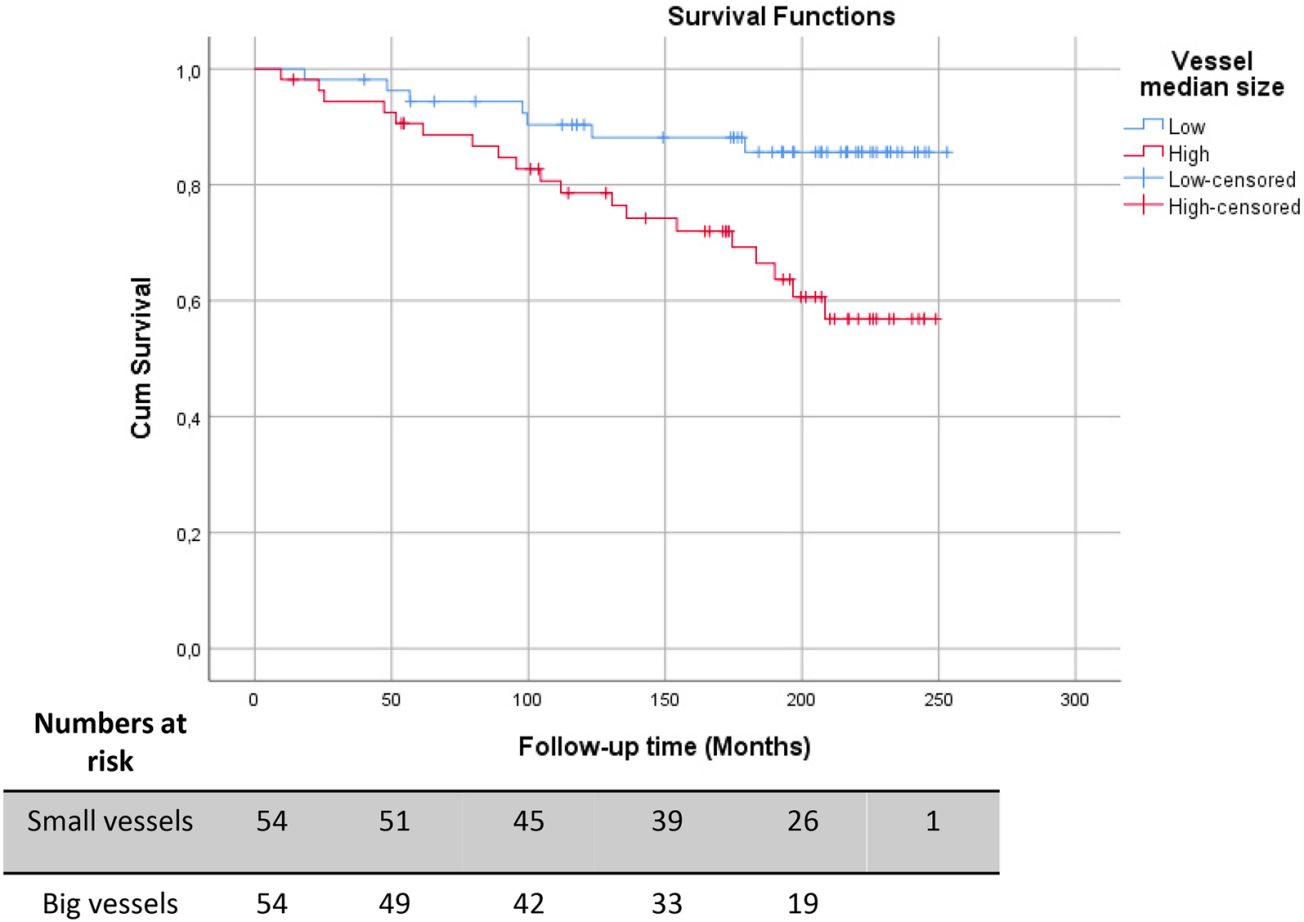
Association of vessel median diameter and cumulative survival (breast cancer-related death as endpoint) in the “whole section” cohort. Vessel median size variable was dichotomized as “low (0–50%)” and “high (> 50%)” group based on the median value. Figure shows Kaplan–Meier curve for cumulative survival (breast cancer-related death as endpoint) represented on the base of the median defined dichotomous values (Log-Rank test, *p* = 0.007; Cox-regression analyses, *p* = 0.01, HR 3.1 (95% CI 1.3–7.4))

Vessel metrics were then analyzed with regard to their associations with cancer-specific survival, following median-based dichotomization of cases into high and low groups. The analyses revealed a statistically significant relationship between large vessel size and shorter disease-specific survival (Fig. 2) (*p* = 0.007 Log-Rank test; *p* = 0.01, HR 3.1 (95% CI 1.3–7.4) Cox-regression analyses). No survival associations were detected for vessel density or fraction of a-SMA-covered vessels using median-based dichotomization, or analyses dividing the cohort into three or four subgroups by tertiles and quartiles (Supplementary Fig. S2).

The univariable analyses were extended to multivariable analyses to explore the potential prognostic independence of vessel size. Multivariable analyses were, therefore, performed including vessel size, age, tumor size, histological grade, lymph node status, ER/PR immunohistochemical status, and molecular subtypes. As shown in Table 1, this analysis identified vessels size as an independent prognostic factor (*p* = 0.01; HR 3.8 (95% CI 1.4–10.5)).

**Table 1:** Vessel median diameter as a prognostic factor in multivariable analysis of the “whole section” cohort

Variables	Multivariable analysis	
	HR (95% CI)	*p* value
Vessel median diameter		
Low	1 (reference)	
High	3.82 (1.39–10.51)	0.01
Age at diagnose		
≤ 60	1 (reference)	
> 60	0.45 (0.16–1.21)	0.11
Tumor size		
≤ 20 mm	1 (reference)	
> 20 mm	0.45 (0.14–1.41)	0.17
Histologic grade		
Grade 1	1 (reference)	
Grade 2	0.63 (0.19–2.15)	0.47
Grade 3	0,77 (0.22–2.66)	0.68
Lymph node status		
N0	1 (reference)	
N1	8.60 (2.98–24.77)	0.001
Estrogen receptor status		
+	1 (reference)	
−	14.66 (1.41–152.487)	0.025
Progesterone receptor status		
+	1 (reference)	
−	2.20 (0.61–7.91)	0.225
HER2 status		
−	1 (reference)	
+	0.22 (0.01–3.76)	0.294
Molecular subtypes		
Luminal A	1 (reference)	
Luminal B/HER2 +	2.42 (0.57–10.25)	0.23
Luminal B/HER2 −	17.61 (0.66–471.6)	0.087
TN	0.16 (0.01–2.17)	0.168

The total of 108 “whole section” cohort cases were included in the regression model. Vessel median size variable was dichotomized as “low (0–50%)” and “high (>50%)” group based on the median value

*P* value are calculated based on Wald test; HR is based on cause-specific Cox-regression model

*HR* hazard ratio, *CI* confidence interval, *HER2* human epidermal growth factor 2, *ER* estrogen receptor, *PR* progesterone receptor, *TN* triple-negative breast cancer

### Prognosis association of vessel size is detected in ER + but not ER − breast cancer

Univariable survival analyses were performed in subsets of breast cancer defined by clinicopathologic characteristics, to further characterize the prognosis associations of vessel size.

**Fig. 3:**
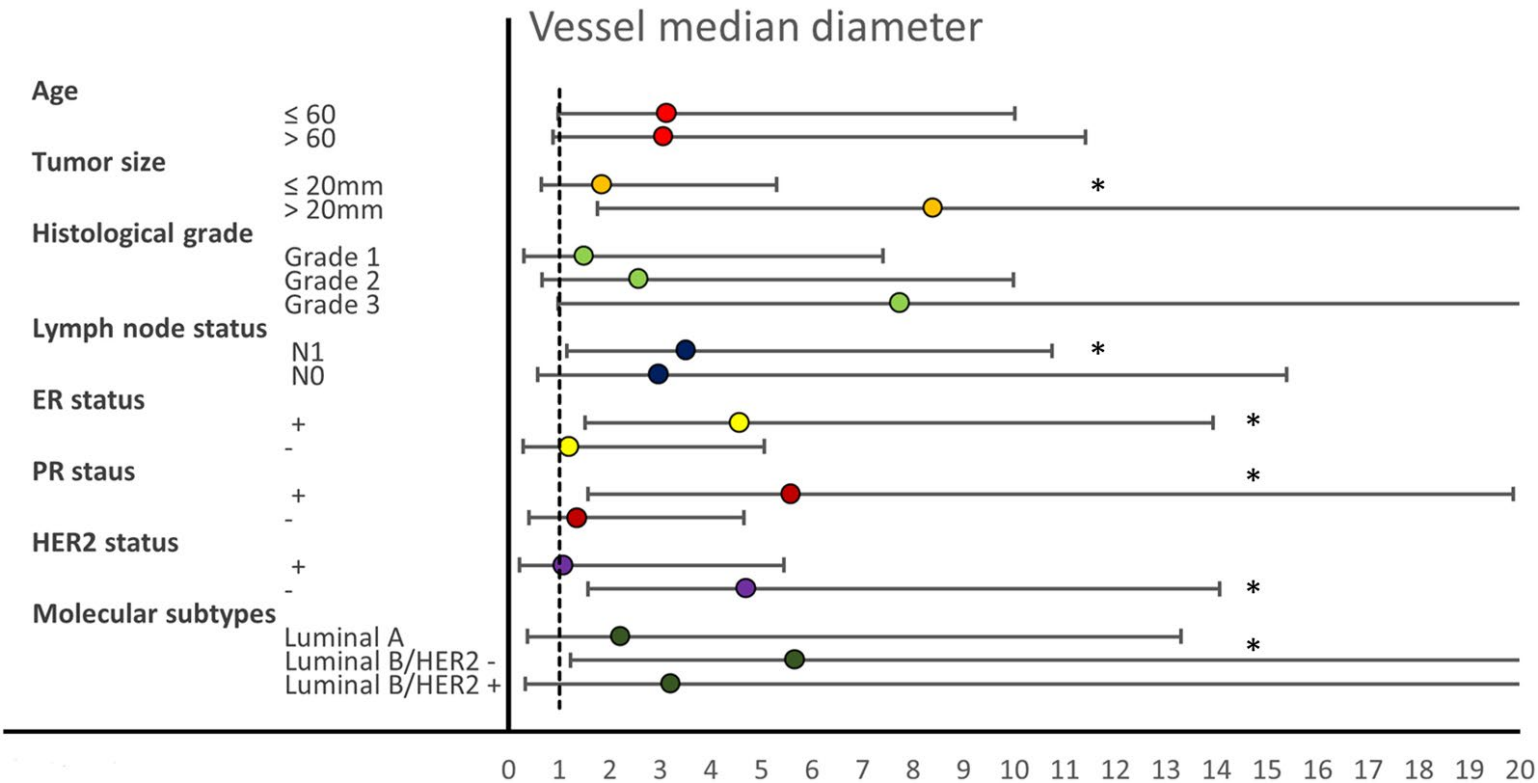
Impact of high vessel diameter on poor survival in different clinicopathologic subsets. HR and confidence intervals presented as the forest plot. *p < 0.05. *p* values are calculated based on Wald test; HR is based on cause-specific Cox-regression model

As shown in Fig. 3 and Supplementary Table S5, the prognosis associations of vessel size showed large variations between breast cancer clinicopathologic sub-groups. The strongest prognostic signals were noted in the groups with lymph node infiltration, large tumors, and in ER +, PR +, HER2-, and Luminal B tumors.

The indications of differential prognosis associations of vessel size in defined subgroups were followed up by additional analyses. Analyses of associations between vessel size and clinicopathologic characteristics in ER + and ER − subgroups did not identify any significant associations (Supplementary Tables S6–S11).

Multivariable survival analyses were performed in the ER + subgroup. As shown in Table 2, large vessel size remained a poor prognosis factor (*p* = 0.01; HR 5.6 (95% CI 1.5–21.5)) in prognostic models that also included patients’ age, tumor diameter, histological grade, lymph node metastasis, PR status, and HER2 status.

**Table 2:** Vessel median diameter as a prognostic factor in multivariable analysis of the “whole section” cohort in ER+ breast cancer subset

Variables	Multivariable analysis	
	HR (95% CI)	*p* value
Vessel median diameter		
Low	1 (reference)	
High	5.63 (1.48–21.48)	0.01
Age at diagnose		
≤ 60	1 (reference)	
> 60	0.36 (0.11–1.15)	0.08
Tumor size		
≤ 20 mm	1 (reference)	
> 20 mm	0.64 (0.16–2.59)	0.53
Histologic grade		
Grade 1	1 (reference)	
Grade 2	0.47 (0.11–2.03)	0.31
Grade 3	0,95 (0.24–3.78)	0.94
Lymph node status		
N0	1 (reference)	
N1	5.89 (1.8–19.28)	0.003
Progesterone receptor status		
+	1 (reference)	
−	2.76 (0.83–9.15)	0.097
HER2 status		
−	1 (reference)	
+	1.88 (0.48–7.35)	0.36

The 86 ER + breast cancer cases of the “whole section” cohort were included in the regression model. Vessel median size variable was dichotomized as “low (0–50%)” and “high (>50%)” group based on the median value

*p* value are calculated based on Wald test; HR is based on cause-specific Cox-regression model

*HR* hazard ratio, *CI* confidence interval, *HER2* human epidermal growth factor 2, *ER* estrogen receptor, *PR* progesterone receptor

### Tissue microarray analyses of additional ER + breast cancer cases confirm independent poor prognosis associations of large vessel size

**Fig. 4:**
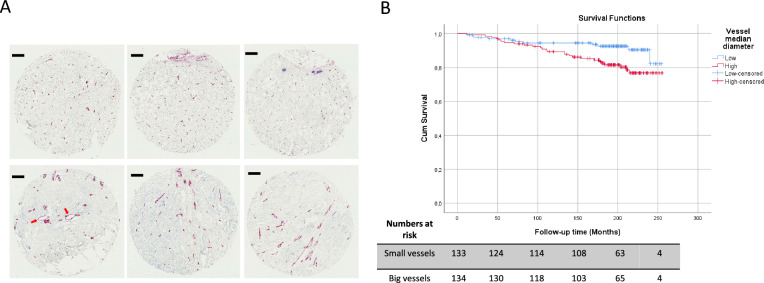
**A** Examples of breast cancer TMA cores with low-vessel median size (upper row) and high-vessel median size (lower row). Each case is represented by three cores belonging to the same case. Blue = a-SMA, red = CD34. The size bar corresponds to 100 μm. Arrows pointing to “Cavitary structures” [61]. **B** Association of vessel median diameter and cumulative survival (breast cancer related death as endpoint) in the ER + breast cancer “TMA” cohort. Vessel median size variable was dichotomized as “low (0–50%)” and “high (> 50%)” group based on the median value. Figures show Kaplan–Meier curve for cumulative survival (breast cancer related death as endpoint) represented on the base of the median defined dichotomous values. (Log-Rank test, *p* = 0.016; Cox-regression analyses (Wald test), *p* = 0.02; HR 2.3 (95% CI 1.1–4.7))

To consolidate findings, additional analyses were performed on tissue microarray samples, containing 3 cores from each tumor, from 267 additional cases of ER + mammography-detected breast cancers. Case characteristics are summarized in Supplementary Table S1. The feasibility of consistent scoring of case-based vessel size on tissue microarray samples was supported by interclass correlation coefficient analyses (ICC) which demonstrated moderate concordance of the vessel median diameter measurements between the cores of each corresponding case (*ICC* = 0.67; 95% CI 0.58–0.75). Example of breast cancers with high and low vessel size, each represented by three cores belonging to the same case, is shown in Fig. 4A.

As in the initial analyses, no significant associations were detected in the ER + cases between vessel size and age, tumor diameter, histological grade, tumor diameter, PR status, or HER2 status (Supplementary Table S12).

As shown in the Kaplan–Meier plot of Fig. 4B, a significant poor prognosis association of large vessel size was also detected in this collection of ER + breast cancer (*p* = 0.016). Results were confirmed by univariable Cox-Regression analyses ((*p* = 0.02; HR 2.3 (95% CI 1.1–4.7)). Furthermore, vessel size remained an independent prognosis marker in multivariable analyses ((*p* = 0.009; HR 2.7 (95% CI 1. 3–5.5)) also including age, tumor diameter, histological grade, lymph node metastasis, PR status, and HER2 status (Table 3).

**Table 3:** Vessel median diameter as a prognostic factor in multivariable analysis of the “TMA” cohort in ER+ breast cancer subset

Variables	Multivariable analysis	
	HR (95% CI)	*p* value
Vessel median diameter		
Low	1 (reference)	
High	2.66 (1.28–5.53)	0.009
Age at diagnose		
≤ 60	1 (reference)	
> 60	1.8 (0.9–3.59)	0.095
Tumor size		
≤ 20 mm	1 (reference)	
> 20 mm	1.92 (0.86–4.26)	0.111
Histologic grade		
Grade 1	1 (reference)	
Grade 2	1.39 (0.64–2.998)	0.41
Grade 3	2.65 (1.00–7.02)	0.05
Lymph node status		
N0	1 (reference)	
N1	3.78 (1.79–7.99)	0.001
Progesterone receptor status		
+	1 (reference)	
−	1.93 (0.86–4.36)	0.112
HER2 status		
−	1 (reference)	
+	0.98 (0.35–2.71)	0.962

The 267 ER+ breast cancer cases of the “TMA” cohort were included in the regression model. Vessel median size variable was dichotomized as “low (0–50%)” and “high (>50%)” group based on the median value

*p* value are calculated based on Wald test; HR is based on cause-specific Cox-regression model

*HR* hazard ratio, *CI* confidence interval, *TMA* tissue microarray, *HER2* human epidermal growth factor 2, *ER* estrogen receptor, *PR* progesterone receptor

## Discussion

In this study, we identified vessel size as a marker of poor prognosis in the ER + subset of breast cancer. Key findings for this claim are the identification of significant associations between this marker and poor prognosis in multivariable analyses of two patient series, one consisting of 108 cases analyzed as whole sections and the other consisting of 267 TMA-based cases of ER + breast cancer.

Some strengths of this study are that the prognostic metric was scored using an automated method increasing objectivity, and also that associations were detected following a simple median-based dichotomization. One limitation, to be addressed and considered in future validation studies, is that the design for the study prevents analyses from clarifying if the survival associations are related to intrinsic tumor aggressiveness or response to subsequently administrated treatment.

Important tasks for future studies include validation in independent cohorts, as well as efforts to systematically identify optimal scoring procedures and cut-offs for this novel biomarker candidate. Regarding methodology for vessel characterization, it is noted that the present study relied on a relatively simple method, used in earlier studies, not specifically resolving the recognized intrinsic problem of scoring properties of three-dimensional vessels on two-dimensional sections. Findings of the present study should motivate continued studies on vessel characteristics in ER + breast cancer, including methods that also allow scoring of features such as fractal dimensions and lacunarity, associated with prognosis in other tumor types [40, 41].

Our study was designed to examine the prognostic value of vessel size, a-SMA perivascular coverage, and vessel density on breast cancer-specific survival. The initial data showed high inter-case heterogeneity of all these three metrics. Correlation analyses pointed out a positive correlation between vessel median size and fraction of a-SMA covered vessels and a negative correlation between vessel median size and vessel density. The latter findings are consistent with active angiogenesis leading to a high number of small vessels. None of our tissue metrics showed any associations with clinicopathologic characteristics in either of the two series. Although perivascular a-SMA coverage has been observed as a prognostic factor in different studies and was associated with poor prognosis in some of them [31–34], in our breast cancer study, this metric showed no association with survival.

To the best of our knowledge, the only earlier study reporting vessel size as a potential prognostic marker performed on breast cancer patients is by Mikalsen et al. [42]. Our observation is in concordance with their finding that large vessels are associated with shorter breast cancer-specific survival. In the study of Mikalsen et al., the authors also focused their attention on the vessel shape complexity as an important factor of survival [42], which was not examined in our present study. To note, our study demonstrated the independent prognostic value of median vessel size when adjusting for age, tumor size, histological grade, HER2 status, progesterone receptor status, and lymph node status in multivariable Cox analysis, not previously shown in the ER + breast cancer subtype.

Vessel density failed to provide independent prognostic value in our study. This vascular feature has been analyzed in multiple earlier studies and has provided divergent results [12, 14–20, 43–46]. Tentative reasons for these discrepancies include the scoring of different tumor sub-regions and variations in methods for vessel scoring [47]. Furthermore, some studies pointed out that vessels with certain qualities can have favorable effects on prognosis [48]. This could explain why our study, among the few others, failed to replicate high microvessel density being a significant factor of poor prognosis, having vessel number taken without considering further their morphological features and also the molecular landscape of the involved endothelium, which has been previously known to have a clinical significance [6, 8, 48–50]. Notably, more consistent signals have been obtained when the density of proliferating vessels has been analyzed [15–20]. Combination analyses also including scoring of proliferating vessels appear highly motivated.

In different clinicopathologic subsets, the impact of vessel diameter on survival in the whole section cohort was significant only in ER/PR + subsets, breast cancers with lymph node metastases, and Lum B/HER2-molecular subtype. It has been known that endothelial cells contain estrogen receptors [51, 52] and that estrogen fulfills its effects as a vascular protector in premenopausal women, aside from other mechanisms, through the reduction of peripheral vascular resistance by increasing vessels’ lumen size [53]. Moreover, it has been reported that ER expression in breast cancer will correlate with higher levels of available estrogen hormone in the breast tissue, which can also be correlated with higher estrogen hormone delivery induced by higher tissue vascularization [54]. Along with this statement, in the study by Lloyd et al., the authors hypothesized that the blood flow arrangements can be the cause of breast cancer cell heterogeneity and that defining vessel characteristics could help predict ER positivity patterns. In this study, the authors reported a strong positive correlation between the vessel size and the positive ER status in breast cancer, observing the mean vessel diameter of ER positive tumors being around twice the mean vessel size in ER negative tumors [54]. Their finding is providing a possible explanation for why our large vessel prognostic signal was enriched particularly in the ER + tumors. Nevertheless, we failed to observe an association between vessel size and ER status in our study. Lloyd et al. also recorded that vessel density was not correlated with the ER status or disease progression [54], which is in concordance with our present findings.

Additional questions that arise from the biological perspective are by which mechanisms large vessels could affect cancer biology, cancer progression, metastasis, and eventually survival. The aberrant vessel anatomy, along with the presence of big and distorted vessels, is known to influence dysfunctional blood flow, perpetuate extravasation of cancer cells, and facilitate metastatic processes, consequentially having a negative effect on overall survival [11]. Angiogenesis has been recognized as an essential piece of the puzzle in the process of tumor metastasis [55]. Although we did not find a correlation between vessel median size and vessel density with the occurrence of lymph node metastasis, previous studies are suggesting an association between vessel size and higher metastatic potential [42, 56–59]. In addition, it has been noted that an increase in the mean vessel diameter is associated with tumor angiogenesis and with larger tumor size [60].

An additional, specific tumor vessel morphology has been described by Senchukova and Kiselevsky in 2014 [61] when they reported the existence of so-called “cavitary structures” (CS), which are stromal structures lined with endothelium and connected with the rest of the tumor vasculature. In our study, we indeed recorded similar structures in patients belonging to the “high vessel median size” group. Additional studies conducted on these structures, defined specific molecular signatures of tumor tissues containing CS, such as high levels of nitric oxide synthase (iNOS), increased synthesis of thrombospondin 4 and high levels of matrix metalloproteinases [55]. In the study from 2015, Senchukova et al. reported two different types of “cavitary structures,” cavitary structure type 1 and type 2 (CS-1, CS-2) [56]. The authors provided evidence that the specific type of these “cavitary structures” (CS-1) is being associated with lymphovascular invasion, the presence of tumor emboli in vessels, and clinically evident metastasis in gastric cancer [56] and breast cancer [58]. Moreover, they reported that the formation of CS-1 was associated with high density of CD68 positive cells in the surrounding stroma and that high density of CD20-positive cells was associated with the formation of CS-2 type [56]. This can suggest biological mechanisms and signaling pathways that might be examined in the future. Further studies investigating the involvement of stromal and immune cells in the formation of CS and big size vessels might prove to be beneficial in detecting pathways and mechanisms involved in angiogenesis, metastasis, and tumor progression and which could be potentially exploited as druggable targets or biomarkers of survival and prognosis.

In summary, our study showed wide variations in the intensity of a-SMA staining across the samples and together with CD34 staining, and revealed high breast cancer heterogeneity regarding vessel size, perivascular a-SMA status, and vessel density. The measured vessel features were not associated with clinicopathological characteristics, but large vessel size was linked to shorter survival. Prognosis association of vessel size was detected in ER +, but not in ER −, breast cancer.

The vessel median size metric has been mostly neglected in tumor vasculature studies and has not received much attention as a possible vascular marker of prognosis. Although it is established knowledge that MVD, pMVD, VPI, and vascular coverage are valid prognostic factors in malignant diseases, our study suggests that the morphology and the size of the vessels, and not only the increase in vascularization, are indicative of prognosis. Our study, thus, suggests that the novel and simple metric of vessel size should be further validated as a biomarker in ER + positive breast cancer and also be explored in other tumor types.

## Supplementary Information

Below is the link to the electronic supplementary material.

Supplementary file1 (DOC 2372 KB)

## Data Availability

Available upon request.
