# Supplementary material for: Vessel size as a marker of survival in estrogen receptor positive breast cancer
Source: Breast Cancer Res Treat. 2023 May 24;200(2):293–304. doi: 10.1007/s10549-023-06974-4 (PMC10241708; doi:10.1007/s10549-023-06974-4)
Supplement: Supplementary file 1 — Supplementary file1 (DOC 2372 KB) [file 10549_2023_6974_MOESM1_ESM.doc]

**Vessel size as a marker of survival in estrogen receptor positive breast cancer**

Vladan Milosevic^1*^, Reidunn J. Edelmann^1,2^, Ingeborg Winge^1^, Carina Strell^3^, Artur Mezheyeuski^3^, Gøril Knutsvik^1,2^, Cecilie Askeland^1,2^, Elisabeth Wik^1,2^, Lars A. Akslen^1,2#^, Arne Östman^1,4#^

^1^Centre for Cancer Biomarkers CCBIO, Department of Clinical Medicine, University of Bergen, Bergen, Norway

^2^Department of Pathology, Haukeland University Hospital, Bergen, Norway

^3^Department of Immunology, Genetics and Pathology, Uppsala University, Uppsala, Sweden

^4^Department of Oncology and Pathology, Karolinska Institutet, Solna, Sweden

^#^ The last two authors contributed equally

*Correspondence to: Vladan Milosevic (v.milosevic@uib.no), ORCID: 0000-0001-6991-6795

**List of supplementary material**

Supplementary Figure S1


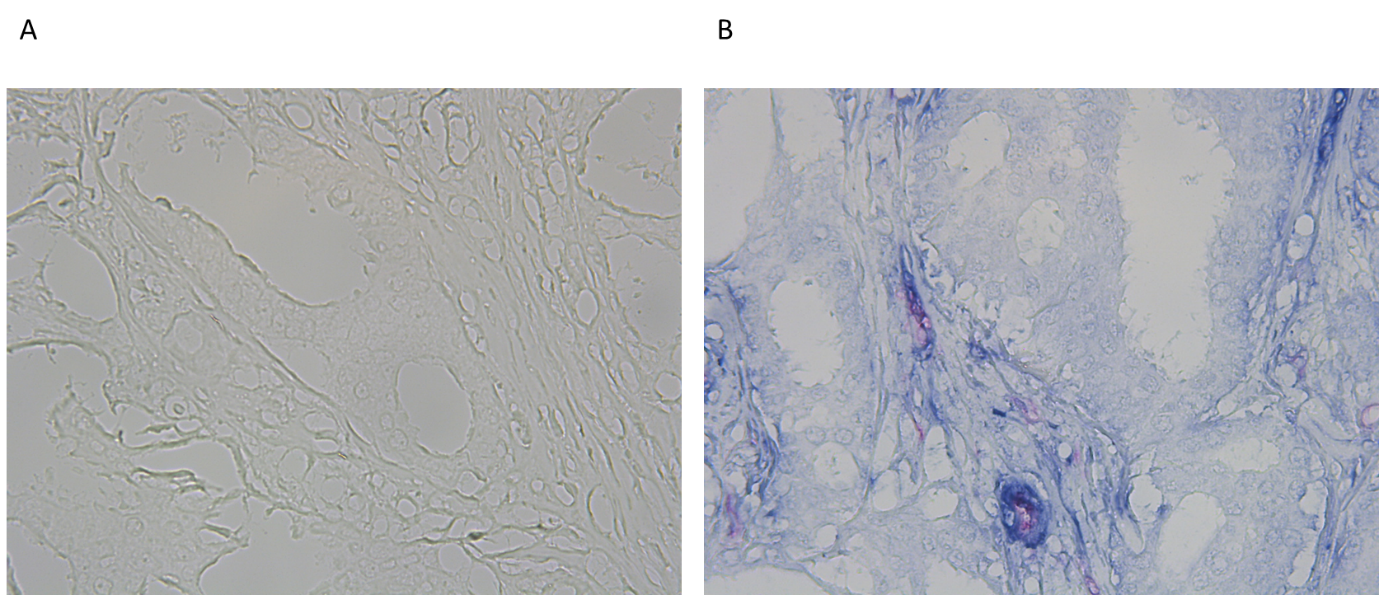


Supplementary Figure S1. Control IHC staining. (A) No primary antibody control staining and (B) positive control dual staining, CD34 (red) / aSMA (blue). Breast cancer tissue, magnification x400.

Supplementary Figure S2

Supplementary Figure S2. Association of vessel density, vessel median diameter and fraction of a-SMA covered vessels and cumulative survival in the “whole section” cohort. Kaplan–Meier curves for cumulative survival calculated on the base of median defined dichotomous values for each of the metrics (left), tertiary values (middle) and quartile values (right). p values are calculated using the Log-Rank-test.

Supplementary Table S1. Comparison of the distribution of clinicopathological characteristics between the Bergen study cohorts presented as large sections and as TMAs.

|  | | | |
| --- | --- | --- | --- |
|  | **Large sections cohort** | **TMA cohort**  **(ER+ subset)** | **p-value** |
| **Patient sample size and median survival time** | | | |
| Total number of patients | 108 (100%) | 267 (100%) |  |
| Median survival time^a^ ± SD (months) | 98.77 ± 60.52 | 105.57 ± 66,52 | 0.632 ^b^ |
| Breast cancer related deaths | 26 (24.1%) | 37(13,9%) | 0.735 ^c^ |
| **Age at diagnosis** | | | |
| Mean ± SD | 60.35 ± 6.04 | 59.96 ± 6.07 | 0.342^d^ |
| Median ± SD | 61 ± 6.04 | 60 ± 6.07 |
| ≤ 60 | 53 (49.1%) | 136 (50,9%) |
| > 60 | 55 (50.9%) | 131 (49.1%) |
| *Missing data* | 0 | 0 |
| **Tumor size, mm** | | | |
| Mean ± SD | 21.27 ± 12.91 | 15.88 ± 9.95 | 0.669^d^ |
| Median ± SD | 18± 12.91 | 13 ± 9.95 |
| ≤ 20mm | 69 (63.9%) | 225 (84.3%) |
| > 20mm | 39 (36.1%) | 42 (15.7%) |
| *Missing data* | 0 | 0 |
| **Histological grade** | | | |
| 1 | 28 (25.9%) | 125 (46.8%) | 0.093^c^ |
| 2 | 50 (46.3%) | 121 (45.3%) |
| 3 | 30 (27.8%) | 21 (7.9%) |
| *Missing data* | 0 | 0 |
| **Lymph node status** | | | |
| N0 | 66 (61.1%) | 201 (75.3%) | 1^d^ |
| N1 | 41 (38%) | 66 (24.7%) |
| Missing data | 1 (0.9%) | 0 |
| **ER status** | | | |
| ER + | 86 (79.6%) | N/A | N/A |
| ER - | 22 (20.4%) |
| *Missing data* | 0 |
| **PR status** | | | |
| PR + | 73 (67.6%) | 217 (81.3%) | 0.441^d^ |
| PR - | 35 (32.4%) | 50 (18.7%) |
| *Missing data* | 0 | 0 |
| **HER2 status** | | | |
| HER2 + | 16 (14.8%) | 29 (10.9%) | 1^d^ |
| HER2 - | 92 (85.2%) | 235 (88%) |
| *Missing data* | 0 | 3 (1,1%) |
| **Molecular subtypes** | | | |
| Luminal A | 28 (25.9%) | N/A | N/A |
| Luminal B/HER2+ | 11 (10.2%) |
| Luminal B/HER2- | 49 (45.4%) |
| HER2 type | 5 (4.6%) |
| TN type | 15 (13.9%) |
| *Missing data* | 0 |

a- Breast cancer related survival, b- p value based on 2-tailed independent t test, c- p value based on Pearson hi square test, d- p-value based on Fisher’s exact test (2-sided).

Abbreviations: TMA- tissue microarray, HER2-human epidermal growth factor 2; ER-estrogen receptor, PR-progesterone receptor, TN-triple negative breast cancer.

Supplementary Table S2: Association of clinicopathological parameters and VD/mm2 as a continuous variable in WS.

| **Spearman correlation of continuous variables** | | |
| --- | --- | --- |
|  | **rho** | **p-value** |
| Age continuous | -0.04 | 0.71 |
| Tumor size continuous | 0.09 | 0.36 |
| **Group comparisons for VD/mm2** | |  |
|  | **Mean (95% CI)** | **p-value**  **(Mann-Whitney test)** |
| **Age at diagnosis** | | |
| ≤ 60 | 403.55 (341.93 – 465.17) | 0.92 |
| > 60 | 439.53 (341.82 – 537.24) |
| **Tumor size, mm** | | |
| ≤ 20mm | 402.55 (325.32-479.78 ) | 0.059 |
| > 20mm | 456.07 (371.46-540.68) |
| **Lymph node status** | | |
| N0 | 416.68 (335.58-497.79) | 0.60 |
| N1 | 417.40 (341.17-493.63) |
| **ER status** | | |
| ER + | 430.17 (364.29-496.05) | 0.43 |
| ER - | 389.45 (265.81-513.08) |
| **PR status** | | |
| PR + | 428.49 (355.17-501.81) | 0.63 |
| PR - | 408.08 (313.67-502.49) |
| **HER2 status** | | |
| HER2 + | 397.59 (271.61-523.58) | 0.93 |
| HER2 - | 426.10 (361.56-490.64) |
|  | **Mean (95% CI)** | **p-value**  **(Kruskal-Wallis test)** |
| **Histological** **grade** | | |
| 1 | 439.86 (280.61-595.11) | 0.47 |
| 2 | 449.27 (371.10-527.44) |
| 3 | 361.29 (279.66-442.92) |
| **Molecular subtypes** | | |
| Luminal A | 522.07 (361.38-682.76) | 0.23 |
| Luminal B/HER2+ | 442.90 (265.50-620.31) |
| Luminal B/HER2- | 371.22 (308.33-434.11) |
| HER2 type | 297.91 (134.96-460.86) |
| TN type | 426.23 (245.25-607.21) |

Abbreviations: WS-whole section, CI-confidence interval, VD-vessel density, HER2-human epidermal growth factor 2; ER-estrogen receptor, PR-progesterone receptor, TN-triple negative breast cancer. The mean and 95% CI were calculated from continuous tissue metrics for each of the patho-clinical groups.

Supplementary Table S3: Association of clinicopathological parameters and vessel median diameter as a continuous variable in WS.

| **Spearman correlation of continuous variables** | | |
| --- | --- | --- |
|  | **rho** | **p-value** |
| Age continuous | 0.07 | 0.46 |
| Tumor size continuous | -0.08 | 0.41 |
| **Group comparisons for vessel median diameter** | |  |
|  | **Mean (95% CI)** | **p-value**  **(Mann-Whitney test)** |
| **Age at diagnosis** | | |
| ≤ 60 | 3.65 (3.49-3.80) | 0.16 |
| > 60 | 3.93 (3.68-4.19) |
| **Tumor size, mm** | | |
| ≤ 20mm | 3.88 (3.66-4.09) | 0.22 |
| > 20mm | 3.64 (3.47-3.82) |
| **Lymph node status** | | |
| N0 | 3.71 (3.54-3.87) | 0.20 |
| N1 | 3.95 (3.65-4.25) |
| **ER status** | | |
| ER + | 3.78 (3.60-3.95) | 0.38 |
| ER - | 3.85 (3.56-4.14) |
| **PR status** | | |
| PR + | 3.79 (3.60-3.98) | 0.62 |
| PR - | 3.79 (3.53-4.05) |
| **HER2 status** | | |
| HER2 + | 3.64 (3.40-3.88) | 0.67 |
| HER2 - | 3.82 (3.65-3.99) |
|  | **Mean (95% CI)** | **p-value**  **(Kruskal-Wallis test)** |
| **Histological** **grade** | | |
| 1 | 3.72 (3.45-3.99) | 0.36 |
| 2 | 3.70 (3.53-3.87) |
| 3 | 4.01 (3.61-4.41) |
| **Molecular subtypes** | | |
| Luminal A | 3.78 (3.42-4.14) | 0.54 |
| Luminal B/HER2+ | 3.72 (3.41-4.02) |
| Luminal B/HER2- | 3.79 (3.55-4.03) |
| HER2 type | 3.48 (2.92-4.04) |
| TN type | 3.97 (3.60-4.33) |

Abbreviations: WS-whole section, CI-confidence interval, HER2-human epidermal growth factor 2; ER-estrogen receptor, PR-progesterone receptor, TN-triple negative breast cancer. The mean and 95% CI were calculated from continuous tissue metrics for each of the patho-clinical groups.

Supplementary Table S4: Association of clinicopathological parameters and fraction of a-SMA covered vessels as a continuous variable in WS.

| **Spearman correlation of continuous variables** | | |
| --- | --- | --- |
|  | **rho** | **p-value** |
| Age continuous | 0.05 | 0.58 |
| Tumor size continuous | -0.03 | 0.78 |
| **Group comparisons for fraction of a-SMA covered vessels** | | |
|  | **Mean (95% CI)** | **p-value**  **(Mann-Whitney test)** |
| **Age at diagnosis** | | |
| ≤ 60 | 0.31 (0.25-0.36) | 0.48 |
| > 60 | 0.35 (0.28 – 0.42) |
| **Tumor size, mm** | | |
| ≤ 20mm | 0.35 (0.29 – 0.41) | 0.42 |
| > 20mm | 0.30 (0.23-0.36) |
| **Lymph node status** | | |
| N0 | 0.31 (0.26-0.36) | 0.51 |
| N1 | 0.36 (0.28-0.44) |
| **ER status** | | |
| ER + | 0.34 (0.29-0.39) | 0.13 |
| ER - | 0.27 (0.18-0.35) |
| **PR status** | | |
| PR + | 0.33 (0.28-0.39) | 0.69 |
| PR - | 0.32 (0.24-0.39) |
| **HER2 status** | | |
| HER2 + | 0.30 (0.19-0.40) | 0.74 |
| HER2 - | 0.33 (0.29-0.38) |
|  | **Mean (95% CI)** | **p-value**  **(Kruskal-Wallis test)** |
| **Histological** **grade** | | |
| 1 | 0.36 (0.26-0.46) | 0.47 |
| 2 | 0.32 (0.27-0.38) |
| 3 | 0.31 (0.21-0.40) |
| **Molecular subtypes** | | |
| Luminal A | 0.35 (0.26-0.44) | 0.69 |
| Luminal B/HER2+ | 0.29 (0.18-0.40) |
| Luminal B/HER2- | 0.35 (0.28-0.42) |
| HER2 type | 0.31 (-0.04-0.67) |
| TN type | 0.26 (0.16-0.35) |

Abbreviations: WS-whole section, CI-confidence interval, HER2-human epidermal growth factor 2; ER-estrogen receptor, PR-progesterone receptor, TN-triple negative breast cancer. The mean and 95% CI were calculated from continuous tissue metrics for each of the patho-clinical groups.

Supplementary Table S5: Impact of high vessel median diameter on survival in different patho-clinical subsets in WS cohort (Cox regression analysis)

|  | **HR (95% CI)** | **p-value** |
| --- | --- | --- |
| **Age at diagnosis** | | |
| ≤ 60 | 3.14 (0.98-10.00) | 0.05 |
| > 60 | 3.07 (0.87-11.40) | 0.09 |
| **Tumor size, mm** | | |
| ≤ 20mm | 1.84 (0.64-5.30) | 0.26 |
| > 20mm | 8.40 (1.76-40.18) | 0.01 |
| **Histological** **grade** | | |
| 1 | 1.50 (0.30-7.40) | 0.62 |
| 2 | 2.58 (0.67-9.98) | 0.17 |
| 3 | 7.75 (0.98-61.35) | 0.05 |
| **Lymph node status** | | |
| N1 | 3.52 (1.15-10.75) | 0.03 |
| N0 | 2.99 (0.58-15.39) | 0.19 |
| **ER status** | | |
| ER + | 4.58 (1.50-13.93) | 0.01 |
| ER - | 1.20 (0.29-5.05) | 0.80 |
| **PR status** | | |
| PR + | 5.59 (1.57-19.86) | 0.01 |
| PR - | 1.36 (0.40-4.65) | 0.63 |
| **HER2 status** | | |
| HER2 + | 1.09 (0.22-5.45) | 0.91 |
| HER2 - | 4.69 (1.57-14.06) | 0.01 |
| **Molecular subtypes** | | |
| Luminal A | 2.22 (0.37-13.29) | 0.38 |
| Luminal B/HER2- | 5.66 (1.22-26.27) | 0.03 |
| Luminal B/HER2+ | 3.21 (0.33-31.70) | 0.32 |

p-value is calculated based on Wald test; HR is based on cause specific Cox regression model.

Abbreviations: WS-whole section, HR-hazard ratio, CI-confidence interval, HER2-human epidermal growth factor 2; ER-estrogen receptor, PR-progesterone receptor.

Supplementary Table S6: Association of clinicopathological parameters and VD/mm2 as a continuous variable in ER+ clinical subset of WS cohort.

| **Spearman correlation of continuous variables** | | |
| --- | --- | --- |
|  | **rho** | **p-value** |
| Age continuous | -0.165 | 0.128 |
| Tumor size continuous | 0.109 | 0.316 |
| **Group comparisons for VD/mm2** | |  |
|  | **Mean (95% CI)** | **p-value**  **(Mann-Whitney test)** |
| **Age at diagnosis** | | |
| ≤ 60 | 449.96 (373.17-526.74) | 0.117 |
| > 60 | 413.75 (308.90-518.60) |
| **Tumor size, mm** | | |
| ≤ 20mm | 407.19 (318.86-495.52) | 0.040 |
| > 20mm | 475.34 (380.76-569.92) |
| **Lymph node status** | | |
| N0 | 432.34 (337.60-527.09) | 0.777 |
| N1 | 409.68 (331.61-487.76) |
| **PR status** | | |
| PR + | 430.91 (355.57-506.26) | 0.914 |
| PR - | 426.66 (283.68-569.64) |
| **HER2 status** | | |
| HER2 + | 422.90 (265.50-620.31) | 0.539 |
| HER2 - | 428.30 (355.91-500.70) |
|  | **Mean (95% CI)** | **p-value**  **(Kruskal-Wallis test)** |
| **Histological** **grade** | | |
| 1 | 451.45 (283.01-619.88) | 0.614 |
| 2 | 450.80 (367.71-533.89) |
| 3 | 338.87 (263.11-414.64) |

Abbreviations: WS-whole section, VD-vessel density, CI-confidence interval, HER2-human epidermal growth factor 2, PR-progesterone receptor. The mean and 95% CI were calculated from continuous tissue metrics for each of the patho-clinical groups.

Supplementary Table S7: Association of clinicopathological parameters and vessel diameter as a continuous variable in ER+ clinical subset of WS cohort.

| **Spearman correlation of continuous variables** | | |
| --- | --- | --- |
|  | **rho** | **p-value** |
| Age continuous | 0.082 | 0.451 |
| Tumor size continuous | -0.126 | 0.246 |
| **Group comparisons for vessel diameter** | |  |
|  | **Mean (95% CI)** | **p-value**  **(Mann-Whitney test)** |
| **Age at diagnosis** | | |
| ≤ 60 | 3.59 (3.42-3.77) | 0.146 |
| > 60 | 3.93 (3.64-4.21) |
| **Tumor size, mm** | | |
| ≤ 20mm | 3.89 (3.64-4.14) | 0.139 |
| > 20mm | 3.55 (3.39-3.72) |
| **Lymph node status** | | |
| N0 | 3.67 (3.48-3.85) | 0.074 |
| N1 | 4.00 (3.62-4.37) |
| **PR status** | | |
| PR + | 3.79 (3.60-3.98) | 0.905 |
| PR - | 3.72 (3.23-4.20) |
| **HER2 status** | | |
| HER2 + | 3.72 (3.41-4.02) | 0.746 |
| HER2 - | 3.78 (3.59-3.98) |
|  | **Mean (95% CI)** | **p-value**  **(Kruskal-Wallis test)** |
| **Histological** **grade** | | |
| 1 | 3.76 (3.47-4.05) | 0.564 |
| 2 | 3.66 (3.48-3.84) |
| 3 | 4.11 (3.39-4.83) |

Abbreviations: WS-whole section, CI-confidence interval, HER2-human epidermal growth factor 2, PR-progesterone receptor. The mean and 95% CI were calculated from continuous tissue metrics for each of the patho-clinical groups.

Supplementary Table S8: Association of clinicopathological parameters and fraction of a-SMA covered vessels as a continuous variable in ER+ clinical subset of WS cohort.

| **Spearman correlation of continuous variables** | | |
| --- | --- | --- |
|  | **rho** | **p-value** |
| Age continuous | 0.05 | 0.65 |
| Tumor size continuous | -0.026 | 0.809 |
| **Group comparisons for fraction of a-SMA covered vessels** | | |
|  | **Mean (95% CI)** | **p-value**  **(Mann-Whitney test)** |
| **Age at diagnosis** | | |
| ≤ 60 | 0.31 (0.25-0.38) | 0.513 |
| > 60 | 0.37 (0.29-0.45) |
| **Tumor size, mm** | | |
| ≤ 20mm | 0.36 (0.29-0.42) | 0.468 |
| > 20mm | 0.32 (0.24-0.40) |
| **Lymph node status** | | |
| N0 | 0.31 (0.25-0.36) | 0.16 |
| N1 | 0.41 (0.31-0.51) |
| **PR status** | | |
| PR + | 0.34 (0.28-0.39) | 0.463 |
| PR - | 0.38 (0.25-0.51) |
| **HER2 status** | | |
| HER2 + | 0.29 (0.18-0.40) | 0.722 |
| HER2 - | 0.35 (0.30-0.41) |
|  | **Mean (95% CI)** | **p-value**  **(Kruskal-Wallis test)** |
| **Histological** **grade** | | |
| 1 | 0.37 (0.27-0.48) | 0.701 |
| 2 | 0.33 (0.27-0.40) |
| 3 | 0.33 (0.19-0.42) |

Abbreviations: WS-whole section, CI-confidence interval, HER2-human epidermal growth factor 2, PR-progesterone receptor. The mean and 95% CI were calculated from continuous tissue metrics for each of the patho-clinical groups.

Supplementary Table S9: Association of clinicopathological parameters and VD/mm2 as a continuous variable in ER- clinical subset of WS cohort.

| **Spearman correlation of continuous variables** | | |
| --- | --- | --- |
|  | **rho** | **p-value** |
| Age continuous | 0.449 | 0.108 |
| Tumor size continuous | 0.262 | 0.365 |
| **Group comparisons for VD/mm2** | |  |
|  | **Mean (95% CI)** | **p-value**  **(Mann-Whitney test)** |
| **Age at diagnosis** | | |
| ≤ 60 | 274.27 (212.61-335.93) | 0.024 |
| > 60 | 591.01 (279.16-902.85) |
| **Tumor size, mm** | | |
| ≤ 20mm | 380.50 (207.54-553.45) | 0.674 |
| > 20mm | 400.19 (184.83-615.55) |
| **Lymph node status** | | |
| N0 | 346.22 (195.72-496.71) | 0.582 |
| N1 | 441.33 (206.91-675.74) |
| **PR status** | | |
| PR + | 342.42 (-70.61-755.44) | 0.779 |
| PR - | 394.15 (257.24-531.06) |
| **HER2 status** | | |
| HER2 + | 297.91 (134.96-460.86) | 0.649 |
| HER2 - | 342.42 (-70.61-755.44) |
|  | **Mean (95% CI)** | **p-value**  **(Kruskal-Wallis test)** |
| **Histological** **grade** | | |
| 1 | 261.30 (-1182.45-1705.05) | 0.754 |
| 2 | 438.07 (108.01-768.13) |
| 3 | 386.92 (221.63-552.21) |

Abbreviations: WS-whole section, VD-vessel density, CI-confidence interval, HER2-human epidermal growth factor 2, PR-progesterone receptor. The mean and 95% CI were calculated from continuous tissue metrics for each of the patho-clinical groups.

Supplementary Table S10: Association of clinicopathological parameters and vessel diameter as a continuous variable in ER- clinical subset of WS cohort.

| **Spearman correlation of continuous variables** | | |
| --- | --- | --- |
|  | **rho** | **p-value** |
| Age continuous | -0.354 | 0.215 |
| Tumor size continuous | 0.205 | 0.482 |
| **Group comparisons for vessel diameter** | |  |
|  | **Mean (95% CI)** | **p-value**  **(Mann-Whitney test)** |
| **Age at diagnosis** | | |
| ≤ 60 | 3.79 (3.44-4.14) | 0.764 |
| > 60 | 3.96 (3.32-4.60) |
| **Tumor size, mm** | | |
| ≤ 20mm | 3.81 (3.42-4.20) | 0.923 |
| > 20mm | 3.91 (3.38-4.44) |
| **Lymph node status** | | |
| N0 | 3.88 (3.52-4.25) | 0.539 |
| N1 | 3.82 (3.27-4.37) |
| **PR status** | | |
| PR + | 3.93 (-5.62-13.49) | 1 |
| PR - | 3.85 (3.55-4.15) |
| **HER2 status** | | |
| HER2 + | 3.48 (2.92-4.04) | 0.140 |
| HER2 - | 3.93 (-5.62-13.49) |
|  | **Mean (95% CI)** | **p-value**  **(Kruskal-Wallis test)** |
| **Histological** **grade** | | |
| 1 | 3.17 (3.05-3.30) | 0.174 |
| 2 | 3.98 (3.26-4.69) |
| 3 | 3.90 (3.52-4.28) |

Abbreviations: WS-whole section, CI-confidence interval, HER2-human epidermal growth factor 2, PR-progesterone receptor. The mean and 95% CI were calculated from continuous tissue metrics for each of the patho-clinical groups.

Supplementary Table S11: Association of clinicopathological parameters and fraction of a-SMA covered vessels as a continuous variable in ER- clinical subset of WS cohort.

| **Spearman correlation of continuous variables** | | |
| --- | --- | --- |
|  | **rho** | **p-value** |
| Age continuous | -0.243 | 0.402 |
| Tumor size continuous | -0.143 | 0.625 |
| **Group comparisons for fraction of a-SMA covered vessels** | |  |
|  | **Mean (95% CI)** | **p-value**  **(Mann-Whitney test)** |
| **Age at diagnosis** | | |
| ≤ 60 | 0.28 (0.15-0.41) | 0.815 |
| > 60 | 0.24 (0.13-0.34) |
| **Tumor size, mm** | | |
| ≤ 20mm | 0.29 (0.14-0.44) | 0.974 |
| > 20mm | 0.24 (0.15-0.33) |
| **Lymph node status** | | |
| N0 | 0.31 (0.16-0.46) | 0.497 |
| N1 | 0.21 (0.13-0.30) |
| **PR status** | | |
| PR + | 0.22 (-0.48-0.91) | 1 |
| PR - | 0.27 (0.18-0.37) |
| **HER2 status** | | |
| HER2 + | 0.31 (-0.04-0.67) | 0.820 |
| HER2 - | 0.22 (-0.48-0.91) |
|  | **Mean (95% CI)** | **p-value**  **(Kruskal-Wallis test)** |
| **Histological** **grade** | | |
| 1 | 0.20 (-0.24-0.64) | 0.876 |
| 2 | 0.26 (0.12-0.41) |
| 3 | 0.28 (0.15-0.41) |

Abbreviations: WS-whole section, CI-confidence interval, HER2-human epidermal growth factor 2, PR-progesterone receptor. The mean and 95% CI were calculated from continuous tissue metrics for each of the patho-clinical groups.

Supplementary Table S12: Association of clinicopathological parameters and vessel diameter as a continuous variable in ER+ clinical subset of TMA cohort.

| **Spearman correlation of continuous variables** | | |
| --- | --- | --- |
|  | **rho** | **p-value** |
| Age continuous | 0.013 | 0.835 |
| Tumor size continuous | -0.009 | 0.882 |
| **Group comparisons for vessel diameter** | |  |
|  | **Mean (95% CI)** | **p-value**  **(Mann-Whitney test)** |
| **Age at diagnosis** | | |
| ≤ 60 | 5.74 (5.51-5.97) | 0.774 |
| > 60 | 5.72 (5.50-5.94) |
| **Tumor size, mm** | | |
| ≤ 20mm | 5.80 (5.63-5.97) | 0.057 |
| > 20mm | 5.33 (4.88-5.77) |
| **Lymph node status** | | |
| N0 | 5.78 (5.60-5.96) | 0.139 |
| N1 | 5.56 (5.22-5.90) |
| **PR status** | | |
| PR + | 5.69 (5.51-5.87) | 0.317 |
| PR - | 5.87 (5.51-6.23) |
| **HER2 status** | | |
| HER2 + | 6.16 (5.63-6.70) | 0.076 |
| HER2 - | 5.68 (5.51-5.84) |
|  | **Mean (95% CI)** | **p-value**  **(Kruskal-Wallis test)** |
| **Histological** **grade** | | |
| 1 | 5.84 (5.60-6.07) | 0.190 |
| 2 | 5.57 (5.33-5.80) |
| 3 | 5.98 (5.38-6.58) |

Abbreviations: TMA-tissue microarray, CI-confidence interval, HER2-human epidermal growth factor 2, PR-progesterone receptor. The mean and 95% CI were calculated from continuous tissue metrics for each of the patho-clinical groups.

Supplementary Table S13. The REMARK criteria checklist

| **Item to be reported** | | **Page no.** |
| --- | --- | --- |
| **INTRODUCTION** | |  |
| 1 | State the marker examined, the study objectives, and any pre-specified hypotheses. | 4 |
| **MATERIALS AND METHODS** | |  |
| *Patients* | |  |
| 2 | Describe the characteristics (e.g., disease stage or co-morbidities) of the study patients, including their source and inclusion and exclusion criteria. | 5 |
| 3 | Describe treatments received and how chosen (e.g., randomized or rule-based). |  |
| *Specimen characteristics* | |  |
| 4 | Describe type of biological material used (including control samples) and methods of preservation and storage. | 5 |
| *Assay methods* | |  |
| 5 | Specify the assay method used and provide (or reference) a detailed protocol, including specific reagents or kits used, quality control procedures, reproducibility assessments, quantitation methods, and scoring and reporting protocols. Specify whether and how assays were performed blinded to the study endpoint. | 6, 7 |
| *Study design* | |  |
| 6 | State the method of case selection, including whether prospective or retrospective and whether stratification or matching (e.g., by stage of disease or age) was used. Specify the time period from which cases were taken, the end of the follow-up period, and the median follow-up time. |  |
| 7 | Precisely define all clinical endpoints examined. | 5 |
| 8 | List all candidate variables initially examined or considered for inclusion in models. |  |
| 9 | Give rationale for sample size; if the study was designed to detect a specified effect size, give the target power and effect size. |  |
| *Statistical analysis methods* | |  |
| 10 | Specify all statistical methods, including details of any variable selection procedures and other model-building issues, how model assumptions were verified, and how missing data were handled. | 8, 9 |
| 11 | Clarify how marker values were handled in the analyses; if relevant, describe methods used for cutpoint determination. |  |
| **RESULTS** | |  |
| *Data* | |  |
| 12 | Describe the flow of patients through the study, including the number of patients included in each stage of the analysis (a diagram may be helpful) and reasons for dropout. Specifically, both overall and for each subgroup extensively examined report the numbers of patients and the number of events. | 9 |
| 13 | Report distributions of basic demographic characteristics (at least age and sex), standard (disease-specific) prognostic variables, and tumor marker, including numbers of missing values. |  |
| *Analysis and presentation* | |  |
| 14 | Show the relation of the marker to standard prognostic variables. |  |
| 15 | Present univariable analyses showing the relation between the marker and outcome, with the estimated effect (e.g., hazard ratio and survival probability). Preferably provide similar analyses for all other variables being analyzed. For the effect of a tumor marker on a time-to-event outcome, a Kaplan-Meier plot is recommended. | 9,  10,  11 |
| 16 | For key multivariable analyses, report estimated effects (e.g., hazard ratio) with confidence intervals for the marker and, at least for the final model, all other variables in the model. |  |
| 17 | Among reported results, provide estimated effects with confidence intervals from an analysis in which the marker and standard prognostic variables are included, regardless of their statistical significance. |  |
| 18 | If done, report results of further investigations, such as checking assumptions, sensitivity analyses, and internal validation. |  |
| **DISCUSSION** | |  |
| 19 | Interpret the results in the context of the pre-specified hypotheses and other relevant studies; include a discussion of limitations of the study. | 12-17 |
|  | Discuss implications for future research and clinical value. |  |
